# An imbalance between RAGE/MR/HMGB1 and ATP1α3 is associated with inflammatory changes in rat brain harboring cerebral aneurysms prone to rupture

**DOI:** 10.1186/s12974-022-02526-7

**Published:** 2022-06-20

**Authors:** Eiji Shikata, Takeshi Miyamoto, Tadashi Yamaguchi, Izumi Yamaguchi, Hiroshi Kagusa, Daiki Gotoh, Kenji Shimada, Yoshiteru Tada, Kenji Yagi, Keiko T. Kitazato, Yasuhisa Kanematsu, Yasushi Takagi

**Affiliations:** Department of Neurosurgery, Graduate School of Biomedical Sciences, Tokushima University, Kuramoto-cho, Tokushima, 770-8503 Japan

**Keywords:** Brain damage, Cerebral aneurysm, SAH, RAGE, HMGB1, MR, ATP1α3

## Abstract

**Background and purpose:**

An aneurysmal subarachnoid hemorrhage is a devastating event. To establish an effective therapeutic strategy, its pathogenesis must be clarified, particularly the pathophysiology of brain harboring intracranial aneurysms (IAs). To elucidate the pathology in brain harboring IAs, we examined the significance of the receptor for advanced glycation end-products (RAGE)/mineralocorticoid receptor (MR) pathway and Na^+^/K^+^-ATPase (ATP1α3).

**Methods:**

Ten-week-old female rats were subjected to oophorectomy as well as hypertension and hemodynamic changes to induce IAs, and were fed a high-salt diet. Brain damage in these rats was assessed by inflammatory changes in comparison to sham-operated rats fed a standard diet.

**Results:**

Six weeks after IA induction (*n* = 30), irregular morphological changes, i.e., an enlarged vessel diameter and vascular wall, were observed in all of the left posterior cerebral arteries (Lt PCAs) prone to rupture. Approximately 20% of rats had ruptured IAs within 6 weeks. In brain harboring unruptured IAs at the PCA, the mRNA levels of *RAGE and MR* were higher, and that of *ATP1α3* was lower than those in the sham-operated rats (*p* < 0.05, each). Immunohistochemically, elevated expression of RAGE and MR, and decreased expression of ATP1α3 were observed in the brain parenchyma adjacent to the Lt PCA, resulting in increased Iba-1 and S100B expression that reflected the inflammatory changes. There was no difference between the unruptured and ruptured aneurysm rat groups. Treatment with the MR antagonist esaxerenone abrogated these changes, and led to cerebral and vascular normalization and prolonged subarachnoid hemorrhage-free survival (*p* < 0.05).

**Conclusions:**

Regulation of the imbalance between the RAGE/MR pathway and ATP1α3 may help attenuate the damage in brain harboring IAs, and further studies are warranted to clarify the significance of the down-regulation of the MR/RAGE pathway and the up-regulation of ATP1α3 for attenuating the pathological changes in brain harboring IAs.

## Background

An aneurysmal subarachnoid hemorrhage (aSAH) is an uncommon and severe subtype of stroke. Although the survival rate after aSAH has increased due to better diagnosis and advances in intensive care, the daily life of aSAH survivors and their ability to work are strongly impacted [1]. To develop more effective therapeutic strategies, the pathogenesis of aSAH must be elucidated.

Previous studies have reported on the inflammatory changes and elevation of vascular degradation molecules in unruptured and ruptured intracranial aneurysms (IAs) [2, 3], and studies using animal models have indicated the possibility of developing treatments using a mineralocorticoid receptor (MR) blocker and an angiotensin type I receptor (AT1R) blockade [4, 5]. Furthermore, these medical treatments were protective against the growth or the rupture of aneurysms in a clinical setting [6]. However, the pathophysiology in brain harboring IAs prone to rupture remains unclear in IA models. To elucidate the pathology in brain harboring IAs prone to rupture, we examined the receptor for advance glycation end-products (RAGE), high-mobility group box 1 (HMGB1), MR, and Na^+^/K^+^-ATPase (ATP1α3) in rats.

Following aSAH, the gene expression and protein levels of RAGE are increased in neurons, suggesting that they are useful prognostic indicators of the outcome after aSAH [7]. RAGE is a multi-ligand receptor that binds to structurally diverse molecules, and is implicated in various chronic inflammatory states [8]. Generation of RAGE-induced reactive oxygen species (ROS) can lead to neuronal death via an imbalance of the redox state [9]. RAGE binds and mediates the cellular response to a range of damage-associated molecular pattern molecules, including HMGB1, S100 calcium-binding protein B (S100B), and DNA. HMGB1 is a highly abundant nuclear protein that interacts with DNA and histones, and regulates key transcription factors, indicating that the silencing of RAGE and HMGB1 during SAH may be a therapeutic option [10–12]. In addition, the gene expression and protein levels of not only HMGB1, but also S100 family members are increased in neurons following SAH, and plasma HMGB1 is a very useful prognostic indicator of the outcome, including death, after aSAH [13].

As a regulator of blood pressure, MR is able to modulate renal sodium handling in response to aldosterone, its principal ligand. The activation of MR with aging contributes to increased blood pressure by regulating the myogenic tone, vasoconstriction, and vascular oxidative stress [14]. In oophorectomized rats fed a high-salt diet (HSD), the elevation of MR and the reduction of a subunit of ATP1α3 were associated with augmented brain damage after ischemia [15], and the down-regulation of ATP1α2 and ATP1α3 was associated with epileptic encephalopathy [16]. ATP1α3 is a subunit of a membrane-bound Na^+^ and K^+^ transporter, which functions by moving three Na^+^ out of the cell in exchange for two K^+^ ions that move into the cell. The predominant α3 subunit is expressed in neurons, and it harnesses the energy of ATP hydrolysis [17].

Unfortunately, it remains unclear how RAGE, MR, HMGB1, and ATP1α3 are expressed and are associated with brain damage in brain harboring IAs prone to rupture. To clarify the expression of RAGE, MR, HMGB1, and pro-inflammatory molecules in brain with IAs prone to rupture, we tested our hypothesis that an imbalance between the brain RAGE/MR pathway and ATP1α3 may be associated with cerebral inflammatory changes in a rat IA model. In addition, we also used the MR antagonist esaxerenone (ESA, CS-3150) to examine the possibility of regulating the expression of RAGE, MR, HMGB1, and pro-inflammatory molecules [18].

## Materials and methods

### Animal preparation

All animal experiments were approved by the ethics committee of the Institute of Tokushima University Graduate School, and were conducted in accordance with the National Institutes of Health Guidelines for the Care and Use of Laboratory Animals. Before any procedure, the rats were anesthetized by 2 to 4% isoflurane inhalation. Female Sprague–Dawley rats were purchased from Charles River Laboratories Japan, Inc. (Tokyo, Japan), and housed in a temperature- and humidity-controlled room (approximately 23 °C and 50%, respectively) under a 12-h light cycle (8 a.m. to 8 *p*.m.) with free access to food and water.

### Induction of IAs

**Fig. 1:**
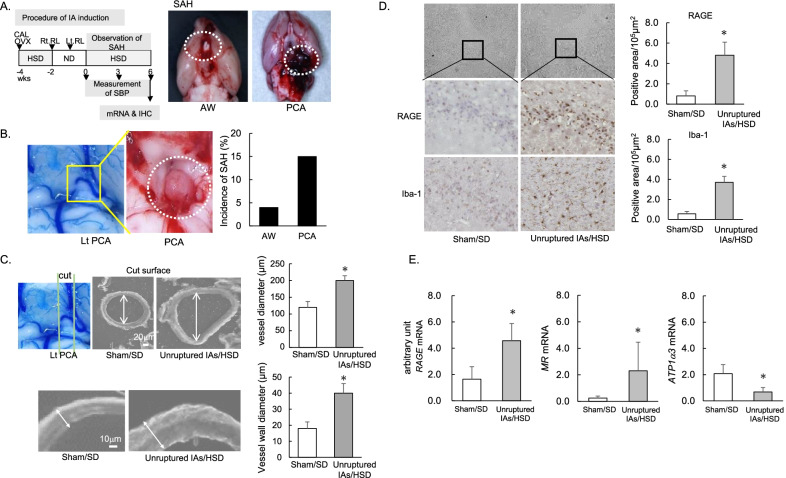
Morphological changes in the left posterior cerebral artery (Lt PCA) prone to rupture and the inflammatory changes in the brain parenchyma adjacent to the Lt PCA. **A** Procedure for the induction of intracranial aneurysms, and representative photographs of SAH due to the rupture of aneurysms at the Lt PCA (right) and the anterior circle of Willis (aCW) (left). *OVX* oophorectomy, *CAL* carotid artery ligation, *Rt RL/Lt RL* right/left posterior renal artery ligation, *SAH* subarachnoid hemorrhage by aneurysm rupture, *HSD* high-salt diet, *SD* standard diet, *SBP* systolic blood pressure, *IHC* immunohistochemistry. **B** Morphological changes with an enlarged blood vessel (left) and a representative rupture (right) at the Lt PCA (dotted circle). The bar graph indicates the incidence of ruptured IAs at the aCW or Lt PCA within 6 weeks (*n* = 30). **C** Sagittal sections of unruptured IAs at the Lt PCA. Arrows indicate the diameter of the vessel lumen and vascular wall. **D** Expression of RAGE and Iba-1 in the brain parenchyma adjacent to the Lt PCA in representative sham-operated rats and rats with unruptured IAs (*n* = 5, each). **E** mRNA levels of RAGE, MR, and ATP1α3 in unruptured IAs (*n* = 6). Data are expressed as the mean ± standard deviation. *p < 0.05 vs. the sham-operated rats by the Student’s *t* test

The IAs were induced as shown in Fig. 1A according to the procedures of a previous study [2]. Ten-week-old female Sprague–Dawley rats (230 to 260 g) were subjected to ligation of the left common carotid, the right external carotid, and the right pterygopalatine arteries to induce hemodynamic stress. Immediately after the ligation, they underwent bilateral oophorectomy (OVX). Subsequently, they received an HSD (8% sodium chloride) for 2 weeks. Then, to induce hypertension, the right posterior renal artery was ligated under a standard diet (SD). One week later, the left posterior renal artery was ligated as well. All surgical procedures were performed under anesthesia. Two weeks after the renal artery ligation, the rats received an HSD. Their blood pressure was recorded using the tail-cuff auto-pickup method (Softron, Tokyo, Japan).

### Observation of SAH and cerebral damage in brain with IAs

To assess the cerebral damage in brain with IAs prone to rupture, we used 30 rats in which IAs were induced; 2 died within the first week, and 3 died within 30 days of the surgery due to surgery-related issues, and they were excluded from this study.

An aneurysm rupture was suspected when the rats died or exhibited abnormal neurological behavior and showed significant weight loss (≥ 30 g/day, approximately 10% of their body weight (BW)). Under a stereomicroscope, we checked and recorded the SAH due to the rupture of an IA at the anterior circle of Willis (aCW) or the left posterior cerebral artery (Lt PCA) prone to rupture [2, 3]. Six weeks after the induction of IAs, diluted Indigo ink was applied to the Lt PCA with a brush to observe the vessel under a stereomicroscope according to a previously reported procedure. [3] The dilatation of the PCA and the angle reflecting atypical structural changes of IAs prone to rupture were observed in all rats. A previous study [3] reported that irregular endothelial damage or aneurysmal changes leading to rupture were observed in all PCAs at 6 weeks after aneurysm induction. In randomly selected rats harboring unruptured aneurysms, the mRNA levels in the brain parenchyma were analyzed and compared to those in sham-operated rats fed an SD (sham/SD). In another set of rats with unruptured or ruptured IAs (*n* = 6 each), the vascular wall at the PCA and the brain tissue adjacent to the PCA were compared immunohistochemically to those of the sham/SD rats (*n* = 6 each).

To assess the therapeutic effects of ESA, an oral nonsteroidal MR blocker with high MR-binding specificity, on brain damage and the IAs, rats were randomly divided into two groups after the induction of IAs. Group 1 served as the vehicle control (VC; 0.5% carboxymethyl-cellulose) group. Group 2 was orally treated with 1 mg/kg/day ESA suspended in VC (*n* = 40 each). During the observation period, three rats in group 1 and five rats in group 2 died due to surgery-related complications, and were excluded from this study.

### Immunohistochemistry

Anesthetized rats were perfused with a 0.9% NaCl solution followed by 4% buffered paraformaldehyde. Their brains were then immersed overnight in 4% buffered paraformaldehyde, then dipped every other day in 10%, 20%, and 30% sucrose phosphate-buffered saline. Using a brain matrix (Bioresearch Center, Nagoya, Japan), each brain was cut into equal 2-mm-thick slices and six serial coronal sections. The brainstem was cut into equal sagittal sections including the Lt PCA prone to rupture. The frozen anterior 4th coronal section and the left sagittal section were embedded in optimal cutting temperature compound for immunohistochemistry.

The anterior 4th coronal section was used for brain tissue staining, and the left sagittal brainstems were used for staining the vessel wall of the Lt PCA. Sections (10 µm in thickness) were sliced with a cryotome (CM 1850; Leica, Nussloch, Germany). After blocking for 30 min in serum-free protein blockade (Dako, Carpinteria, CA, USA), each section was incubated with primary antibody against RAGE, MR, ATP1α3, HMGB1, S100B (Abcam, Cambridge, UK), NFκB (BD Biosciences, NJ, USA), or Iba-1 (Wako, Osaka, Japan) diluted with Can Get Signal Immunostain solution (1:100 dilutions; Toyobo, Osaka, Japan) overnight at 4 °C. Sections without primary antibody were used as the negative controls. Some sections were then incubated for 1 h at room temperature with fluorescein-conjugated secondary antibody (Alexa Fluor 594; Molecular Probes, CA, USA) in Can Get Signal Immunostain solution (1:1000 dilution; Toyobo), and mounted with fluorescence mounting medium (Dako). The other sections were subjected to 3,3'-diaminobenzidine staining. All sections were observed with a microscope-equipped image analyzer (KEYENCE BZ-X710, Keyence, Osaka, Japan).

### Quantitative real-time polymerase chain reaction (qRT-PCR) assay

For the qRT-PCR assay, rats were perfused with a 0.9% NaCl solution. The brains were cut into equal 2-mm-thick slices and the left bottom of the temporal lobe in the anterior 4th coronal section was used for the qRT-PCR assay. Tissue samples from the proximal Lt PCA (between the basilar bifurcation and the posterior communicating artery (PcomA)) were collected for the mRNA assay of the blood vessels. We also collected six random tissue samples from the left bottom of the temporal lobe in the anterior 4th coronal section and from the proximal Lt PCA (between the basilar bifurcation and the PcomA) of sham-operated-, VC-, and ESA-treated rats.

Total RNA was extracted using the MagNA Pure RNA isolation kit (Roche, Tokyo, Japan) and placed in a MagNA Lyser (Roche). We used Transcriptor Universal cDNA Master (Roche) for the reverse transcription of total RNA to cDNA, and a LightCycler 2.0 (Roche Diagnostics, Tokyo, Japan) for qRT-PCR assays. Light Cycler Fast Start DNA Master and SYBR Green I (Roche Diagnostics) were used for glyceraldehyde 3-phosphate dehydrogenase *(GAPDH), MR, RAGE, HMGB1, and ATP1α3*. The primers were: for *GAPDH*, forward (F), 5’-TAC ACT GAG GAC CAG GTT G-3’, reverse (R), 5’-CCC TGT TGC TGT AGC CAT A-3’; for MR, F, 5’-CCT TCC CAC CTG TCA ATA C-3’, R, 5’-GAA GCC TCA TCT CCA CAC A-3’; for RAGE, F, 5’-TCA ACA TCA GGG TCA CAG AAA C-3’, R, 5’-CAA TGA GCA GAG AGC GGC TA-3’; for HMGB1, F, 5’-GAG ATC CTA AGA AGC CGA GA-3’, R, 5’-CTT CCT CAT CCT CTT CAT CC-3’; and for ATP1α3, F, 5’-AAG GAG CAG CCT CTG GAT-3’, R, 5’-GTT CCT CCG GCA GGT AGT AA-3’. The PCR conditions were 95 °C for 10 min followed by 40 cycles at 95 °C for 10 s, 60 °C for 10 s, and 72 °C for 8 s. We subjected the samples from each group to two independent qRT-PCR assays. The results were quantified after normalization to the expression level of *GAPDH* mRNA.

### Water-free sodium accumulation in brain

Based on our previous study [15], sham-operated rats fed an SD (sham/SD) or HSD (sham/HSD), oophorectomized HSD rats (OVX/HSD), and rats subjected to OVX and bilateral posterior renal ligation (OVX/HT/HSD) were used. After perfusion with a 0.9% NaCl solution, the brain was cut into equal 2-mm-thick slices. The anterior 4th coronal sections from each group (*n* = 6) were assayed and the brain water and Na^+^ contents were calculated.

After measuring the wet weight (Ww), the brain was dried in a freeze dryer (EYELA FDU-1000, Rikakikai, Tokyo, Japan) for 24 h, and the dry weight (Dw) was determined. The brain water content (ml/g wet tissue weight) was calculated using the formula: brain water content = (Ww – Dw) (ml) / Ww (g wt). To measure the brain Na^+^ content, the dried brain was homogenized with 1 ml of saline (153 mEq) on ice for 1 min using a pulse ultrasonicator (Tomy Seiko, Tokyo, Japan). After centrifugation at 3000 G (Tomy-MX300) the supernatant was collected, and the Na^+^ was measured using an ion-selective electrode method (SRL Inc., Tokyo, Japan). The brain content of Na^+^ was calculated using the formula: [Na^+^] (mmol/g wt) = {[supernatant Na^+^] (mmol) – 153} / (1 + dry Wt (g wt)) × dry Wt (g wt).

### Statistical analysis

All data are presented as the mean ± standard deviation. We first examined the normality of continuous variables based on the results of the Shapiro–Wilk test. Inter-group differences were examined with the Student’s *t* test. Multiple groups were analyzed with the Dunnett’s test. SAH-free survival from the Kaplan–Meier curve was analyzed by the log-rank test. We applied repeated measure analysis of variance. Differences with a *p* < 0.05 were considered to be statistically significant. Statistical analyses were performed using JMP 13.2 (SAS Institute Inc., Tokyo, Japan).

## Results

### The up-regulation of RAGE/MR and the down-regulation of ATP1α3 are associated with inflammatory changes in brain harboring unruptured IAs

We previously reported that more than half of our aneurysm model rats developed ruptures during the 12-week observation period [2, 3]. Some of the aneurysm ruptures were observed within 6 weeks after aneurysm induction. Based on these findings, in the present study, we examined the brain damage in the rats with aneurysmal changes at 6 weeks after aneurysm induction.

In all rats at 2 weeks after the induction of IAs, the blood pressure was elevated (> 200 mmHg), and it remained high for 6 weeks, as has been reported previously [2, 3]. Approximately 20% of the rats had ruptured IAs within 6 weeks. Ruptures at the Lt PCA (Fig. 1A) were observed earlier and were more common than ruptures at the aCW (Fig. 1B). Compared to the sham/SD rats, unruptured aneurysms at the PCA showed enlarged vessel diameters and vascular walls with areas of bulging (Fig. 1C).

In the brain parenchyma adjacent to the Lt PCA prone to rupture, the expression levels of RAGE and Iba-1 were significantly higher in the rats with unruptured IAs than in the sham-operated rats (*n* = 6, each; Fig. 1D). The mRNA levels of *RAGE and MR* were also elevated, whereas the mRNA level of *ATP1α3* was significantly decreased in the brains of the rats with unruptured IAs when compared to the sham/SD rats (Fig. 1E). The up-regulation of *RAGE/MR* and the down-regulation of *ATP1α3* may be associated with the inflammatory changes in brain harboring unruptured IAs.

### Elevated expression of RAGE, MR and HMGB1, and decreased expression of ATP1α3 in the brain parenchyma adjacent to the Lt PCA harboring unruptured and ruptured IAs

Next, we further assessed the relationships between the expression of RAGE, MR, HMGB1, and ATP1α3 and the cerebral inflammatory changes in the rat brain harboring ruptured and unruptured IAs.

**Fig. 2:**
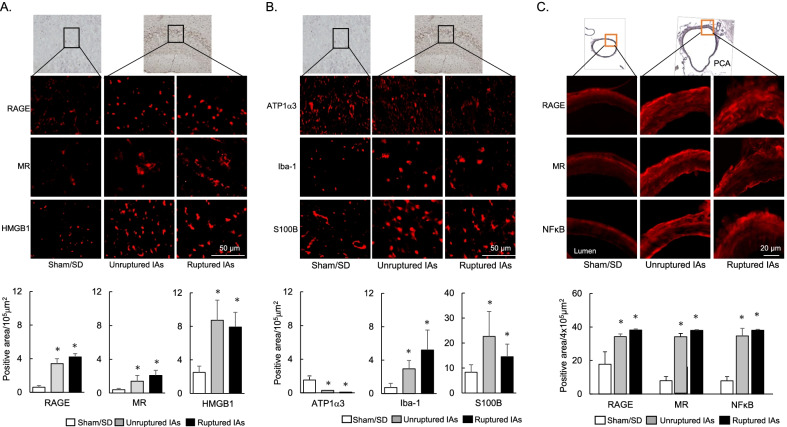
Expression of RAGE, MR, HMGB1, and ATP1α3 in brain parenchyma with inflammatory changes, and the vascular expression of RAGE, MR, and NFκB in rats harboring IAs prone to rupture. **A** Elevated expression levels of RAGE, MR, and HMGB1 in the brain parenchyma adjacent to the Lt PCA prone to rupture. **B** Reduced expression level of ATP1α3 and the increased expression levels of Iba-1 and S100B in the brain parenchyma adjacent to the Lt PCA. **C** Vascular expression levels of RAGE, MR, and NFκB at the Lt PCA. At 6 weeks after IA induction or at the time of rupture within 6 weeks, the area adjacent to the Lt PCA prone to rupture (**A**, **B**) and the vascular wall (**C**) were harvested. Data are expressed as the mean ± standard deviation (*n* = 5). *p < 0.05 vs. the sham/SD rats by the Dunnett’s test

Compared to the sham/SD rats, the expression levels of RAGE, MR, and HMGB1 in the rats with unruptured and ruptured aneurysms were significantly increased in the brain parenchyma adjacent to the Lt PCA with unruptured and ruptured IAs, but not in other areas (Fig. 2A). In contrast, the expression level of ATP1α3 was significantly decreased (Fig. 2B), and was accompanied by increased expression levels of Iba-1 and S100B, reflecting the inflammatory response (Fig. 2B). We also assessed the expression levels of RAGE, MR, and NFκB at the Lt PCA in the rats harboring unruptured or ruptured IAs; compared to the Lt PCA in the sham/SD rats, the expression levels of RAGE, MR, and NFκB were significantly elevated in the unruptured and ruptured PCA (Fig. 2C).

### ***The up-regulation of RAGE and the down-regulation of ATP1α3 are associated with elevated brain water-free Na***^+^***content in OVX-hypertensive rats***

**Fig. 3:**
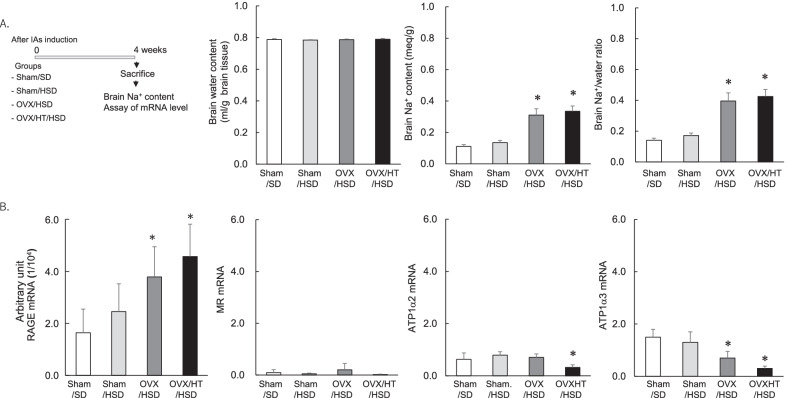
Elevated mRNA level of RAGE and the decreased mRNA level of ATP1α3 associated with the brain water-free sodium accumulation in OVX/HT/HSD rats. **A** Brain water and Na^+^ accumulation in the oophorectomized hypertensive rats fed a high-salt diet (OVX/HT/HSD) and the sham-operated rats fed a standard diet (sham/SD). **B** Elevated mRNA level of RAGE was inversely correlated with the decreased mRNA levels of *ATP1α2 and ATP1α3*. In the brain at 4 weeks after IA induction, the brain water and Na^+^ accumulation and the mRNA levels of *RAGE, MR, ATP1α2, and ATP1α3* were analyzed. Data are expressed as the mean ± standard deviation (*n* = 6). *p < 0.05 vs. the sham/SD rats by the Dunnett’s test

We previously demonstrated that in OVX rats fed an HSD (OVX/HSD), the elevation of the brain Na^+^/water ratio was associated with the down-regulation of ATP1α3, and the up-regulation of MR under normal blood pressure exacerbated the brain damage after cerebral ischemia. In this study, elevation of the brain Na^+^/water ratio was observed in the OVX/HT/HSD rats without hemodynamic changes and in the OVX/HSD rats (Fig. 3). These rats had a significantly elevated mRNA level of RAGE and decreased mRNA levels of astrocytic *ATP1α2* and neuronal *ATP1α3* in the brain, suggesting that the elevation of the brain Na^+^/water ratio is associated with the imbalance between RAGE and ATP1α3, resulting in brain damage.

### Treatment with ESA normalized the enlarged vessel diameter and vascular wall, and attenuated the expression of RAGE and MR at the PCA without detrimental effects

**Fig. 4:**
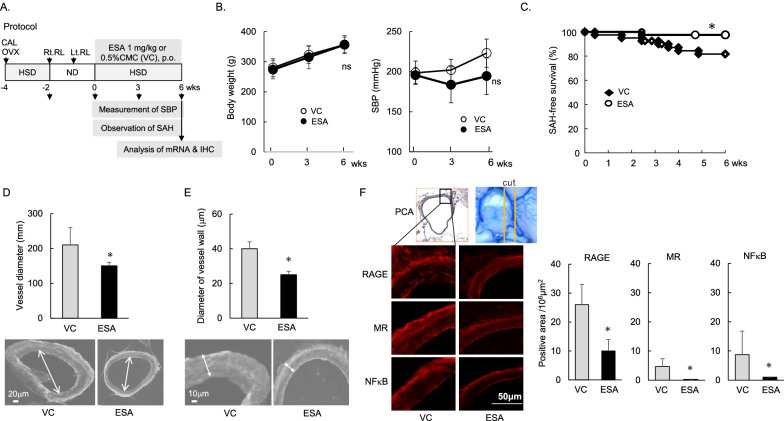
Treatment with esaxerenone (ESA) improved SAH-free survival without affecting body weight, and attenuated the vascular enlargement in the rupture-prone PCA. **A** Protocol for ESA treatment in rats harboring IAs prone to rupture. **B** Effects of ESA on the systolic blood pressure and body weight. **C** Effects of ESA on the SAH-free survival. **D**, **E** Effects of ESA on the vessel diameter and vascular wall at the Lt PCA in comparison to treatment with VC (*n* = 6). **F** Expression levels of RAGE, MR, and NFκB at the Lt PCA treated with ESA in comparison to that treated with VC (*n* = 5). Data are expressed as the mean ± standard deviation. *p < 0.05 vs. the sham-operated rats by the Student’s *t* test

Pro-inflammatory cytokines, including RAGE expression are associated with the activation of NFκB [19]. To regulate the RAGE, MR, and NFκB pathway, we orally treated our aneurysm model rats for 6 weeks with a mineral corticoid receptor blocker, ESA, at 1 mg/kg (Fig. 4A), based on previous studies [18, 20]. ESA-treated rats showed a tendency for decreased blood pressure without changes in BW (Fig. 4B). ESA significantly prolonged the SAH-free survival (Fig. 4C), normalized the enlarged vessel diameter and vascular wall of the PCA (Fig. 4D, E), and lowered the vascular expression of RAGE, MR, and NFκB (Fig. 4F).

### Treatment with ESA abrogated the imbalance between the RAGE/MR pathway and ATP1α3, which is related to inflammatory changes in rat brain harboring unruptured IAs

**Fig. 5:**
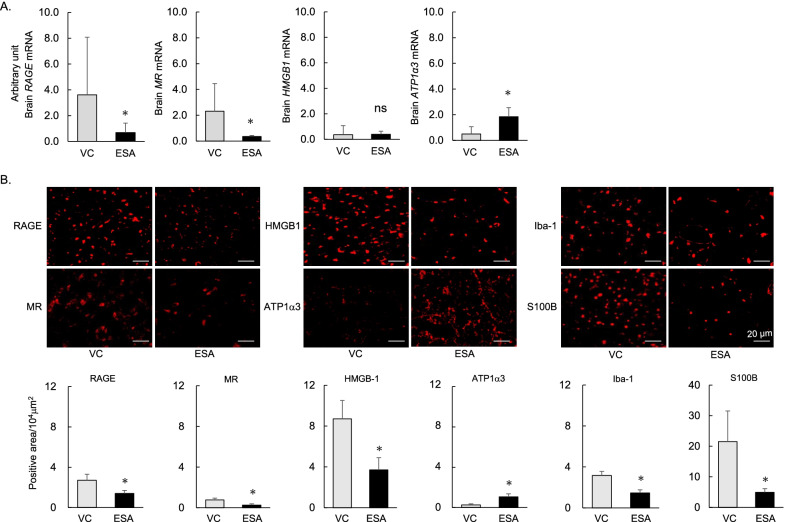
Treatment with ESA abrogated the inflammatory changes related to the elevated levels of RAGE, MR and HMGB1, and the decreased level of ATP1α3 in rat brain harboring unruptured aneurysms. **A** Down-regulated mRNA levels of *RAGE and MR,* and the up-regulated mRNA level of *ATP1α3* in the brain parenchyma adjacent to the Lt PCA in rats treated with ESA (*n* = 6). **B** Treatment with ESA decreased the expression levels of RAGE, MR, and HMGB1 along with decreases in S100B and Iba-1, and increased the expression level of ATP1α3 in the rat brain harboring unruptured IAs (*n* = 5). Data are expressed as the mean ± standard deviation. *p < 0.05 vs. VC by the Student’s *t* test. ns: not significant

Finally, we assessed the effects of ESA on the expression levels of RAGE, MR, HMGB1, and ATP1α3 in brain harboring IAs prone to rupture at the Lt PCA. ESA significantly decreased the mRNA levels of *RAGE and MR*, but not *HMGB1*, and significantly increased the mRNA level of *ATP1α3* when compared to the VC (Fig. 5A). Immunohistochemically, ESA treatment reduced the expression levels of RAGE, MR and HMGB1, and increased the expression level of ATP1α3, resulting in the low expression of S100B and Iba-1 (Fig. 5B). These findings suggest that treatment with ESA may help prevent both cerebral and vascular damage.

## Discussion

The deleterious effects of brain harboring IAs prone to rupture have not been addressed in experimental animals. Here, we documented for the first time the up-regulation of the RAGE/MR pathway and the down-regulation of ATP1α3, which are associated with the inflammatory changes in the brain parenchyma of rats harboring IAs prone to rupture.

After aneurysm induction, approximately 20% of the rats had ruptured IAs. The remaining rats showed morphological changes with an enlarged vessel at the Lt PCA prone to rupture. The vessels had an enlarged diameter and vascular wall, and showed irregular endothelial morphological changes, including areas of bulging, when compared to the sham/SD rats. In the brain parenchyma adjacent to the Lt PCA prone to rupture, the up-regulation of the RAGE/MR pathway and the down-regulation of ATP1α3 were observed along with increased expression of S100B and Iba-1, reflecting the brain damage.

Treatment with ESA normalized the enlarged diameter of the vessel and vascular wall, and reduced the expression levels of RAGE, MR, and NFκB. In addition, it improved the imbalance between the RAGE/MR pathway and ATP1α3, and abrogated the expression of S100B and Iba-1 in the brain, resulting in prolonged SAH-free survival. These findings indicate that a therapeutic strategy targeting the RAGE/MR pathway and ATP1α3 may be a promising approach for not only inhibiting brain damage, but also prolonging SAH-free survival.

RAGE belongs to a family of cell adhesion molecules, and is considered a key receptor in the inflammation axis and a potential contributor to neurodegeneration. Gasparotto et al. [21] reported a role for RAGE in the establishment of a neuroinflammation–neurodegeneration axis that develops as a long-term response to systemic inflammation. Consistent with this finding, we found that the elevated expression levels of RAGE, MR, and HMGB1 in the brain parenchyma adjacent to the Lt PCA prone to rupture were associated with increased levels of Iba-1- and S100B-positive cells, reflecting the inflammatory changes in this area.

On the other hand, we found a decrease in the neuronal subtype of ATP1α3 in the same area. The activity of ATP1α3, a neuronal subtype of the Na^+^ efflux pump, is reduced or insufficient for maintaining an adequate ionic balance during and after episodes of epilepsy and brain injury [16, 22]. We previously reported in OVX rats fed an HSD that the increased expression levels of RAGE, MR, HMGB1, TNFα and TLR9, and reduced expression level of ATP1α3 in the cerebral cortex were associated with expansion of the cortical infarction [23], which was associated with water-free Na^+^ accumulation (Na^+^/water ratio) in the brain. Consistent with these findings, we observed that the down-regulation of ATP1α3 was associated with the accumulation of water-free Na^+^ in the brains of OVX/HT/HSD rats without hemodynamic changes. The down-regulation of ATP1α2 and ATP1α3 was inversely correlated with the up-regulation of RAGE. Although we could not assess the functional activity of ATP1α2 or ATP1α3, the reduced expression levels may reflect deficient ATP1α2 and ATP1α3 activity, leading to brain damage.

Wang et al. [14] reported that MR enhances AT1R signaling in the paraventricular nucleus of hypertensive rats. In the presence of high levels of sodium ions, circulating aldosterone may be increased via MR and AT1R in the sub-frontal area and local aldosterone production in hypothalamic nuclei. AT1R activated by angiotensin II can activate various intracellular protein kinases, such as mitogen-activated protein kinase family proteins and protein kinases C and B, leading to cellular degradation and the production of extracellular HMGB1 [24, 25]. The expression of ligands, such as HMGB1 and S100B, was much higher in the presence of cerebral infarction. This suggests that RAGE-induced ROS generation eliciting an imbalance in the redox state may lead to neuronal death, resulting in the secretion of HMGB1 by the damaged cells. Unexpectedly, the expression levels of RAGE and HMGB1 were not significantly different between the ruptured and unruptured IA groups in this study. Therefore, we could not explain why early brain injury is severe in ruptured IAs in the clinical setting.

The ROS directly compromise control of the vascular tone by reducing the bioavailability of the vasodilator nitric oxide [26], thereby eliciting pro-inflammatory effects. The binding of RAGE to circulating ligands, such as advanced glycation end-products and S100B, leads to the generation of ROS and activates NFκB, resulting in the up-regulation of adhesion molecules for circulating monocytes as well as further up-regulation of RAGE itself [26, 27]. Thus, the high expression levels of RAGE and NFκB may be associated with pro-inflammatory effects in the rupture-prone PCA wall.

**Fig. 6:**
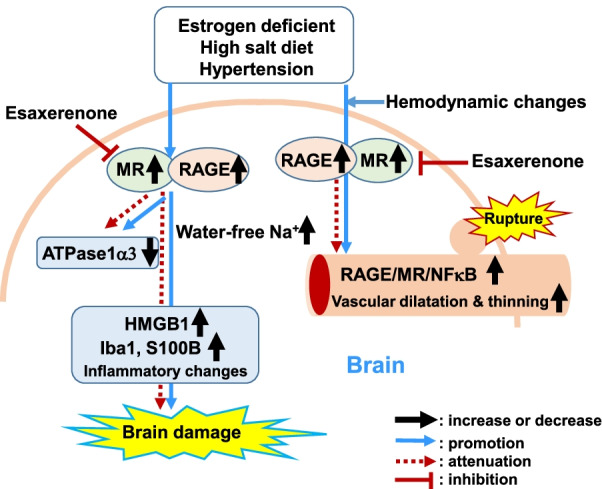
Schematic diagram of damage to rat brain harboring intracranial aneurysms prone to rupture and the effects of ESA

On the other hand, MR binding to aldosterone and angiotensin II promotes oxidative stress, inflammation, cell proliferation, cell migration, and extracellular matrix production, thereby promoting vasoconstriction and atherosclerosis [28, 29]. In addition, a previous study demonstrated that RAGE, GTP-bound Rac-1 and MR were co-localized in the podocytes of DOCA mice. Aldosterone (Aldo) increased RAGE gene expression in murine podocytes, and stimulated MR, which were blocked by RAGE-aptamer, indicating crosstalk between the AGE–RAGE axis and Aldo-MR system in MR-associated renal diseases [30]. In line with these findings, our study demonstrated the elevation of these molecules associated with brain inflammatory changes and the enlargement of the vascular wall prone to rupture. However, treatment with ESA, an MR antagonist, attenuated inflammatory changes in the brain and normalized the enlarged cerebral vascular wall (Fig. 6). Taken together, our findings suggest that the elevated levels of RAGE and MR may play a role in the pathological condition of IAs in the brain as well as in the vascular wall.

Previously, we reported that in the postoperative management after aSAH, infusion with a physiological amount of water and sodium significantly improved the neurological prognosis of patients at discharge when compared to a high-sodium infusion [31]. Given that a high brain Na^+^ content was associated with the down-regulation of ATP1α3 and the up-regulation of RAGE, the improved outcome due to management at the optimal physiological level of sodium may be partly attributed to the normalization of the RAGE/MR pathway and ATP1α3.

### Limitations

Our study has some limitations. We could not trace the vascular conditions at various timepoints after IA induction in the unruptured IAs until they ruptured. Although we expected the expression levels of HMGB1 to be higher in ruptured than unruptured aneurysms, there was no significant difference. Differences in the timing of sacrifice (3 to 4 weeks and 6 weeks after aneurysm induction) may have affected this finding. Although we tried to identify ruptured IAs as soon as possible, they were identified at various timepoints after rupture, resulting in no difference between the unruptured and ruptured IAs. Nonetheless, our findings highlight the importance of the down-regulation of the expression of RAGE, MR and pro-inflammatory molecules, and the up-regulation of the expression of ATP1α3 for preventing the rupture of IAs and improving the prognosis.

## Conclusions

In the brains of the rats with experimental IAs prone to rupture, we demonstrated for the first time that an imbalance between the RAGE/MR pathway and ATP1α3 is associated with the inflammatory changes in the brain parenchyma adjacent to the Lt PCA containing IAs prone to rupture. These changes were abrogated by the MR antagonist ESA, suggesting the significance of regulating the imbalance between the RAGE/MR pathway and ATP1α3. Further studies are required to elucidate the characteristics of the imbalance between the RAGE/MR pathway and ATP1α3, and the usefulness of ESA in a clinical setting.

## Data Availability

The data sets used and/or analyzed during the current study are available from the corresponding author on reasonable request.

## Abbreviations

aSAH: Aneurysmal subarachnoid hemorrhage
RAGE: Receptor for advanced glycation end-products
ROS: Reactive oxygen species
OVX: Ovariectomy
ESA: Esaxerenone
PCA: Posterior cerebral artery
MR: Mineralocorticoid receptor
HMGB1: High mobility group box 1
aCW: Anterior circle of Willis
S100B: S100 calcium-binding protein B
